# Tryptase levels in children presenting with anaphylaxis to the Montreal Children’s Hospital

**DOI:** 10.1186/1710-1492-10-S1-A66

**Published:** 2014-03-03

**Authors:** Michelle Halbrich, Ann Clarke, Sebastian La Vieille, Harley Eisman, Reza Alizadehfar, Joseph Lawrence, Chris Mill, Judy Morris, Moshe Ben-Shoshan

**Affiliations:** 1 Division of Paediatric Allergy and Clinical Immunology, Department of Paediatrics, McGill University Health Centre, Montreal, Quebec, Canada; 2 Division of Clinical Epidemiology, Department of Medicine, McGill University Health Centre, Montreal, Quebec, Canada; 3 Division of Allergy and Clinical Immunology, Department of Medicine, McGill University Health Centre, Montreal, Quebec, Canada; 4 Food Directorate, Health Canada, Ottawa, Ontario, Canada; 5 Montreal Children’s Hospital, Emergency Department, McGill University Health Centre, Montreal, Quebec, Canada; 6 Departments of Epidemiology and Biostatistics, McGill University, Montreal, Quebec, Canada; 7 Department of Emergency Medicine, Hôpital du Sacré-Coeur de Montréal, Université de Montréal, Québec, Canada

## Rationale

There are little data on the role of tryptase in the diagnosis of anaphylaxis. We aimed to assess the sensitivity of elevated tryptase levels (>11.4) as defined in the current medical literature to diagnose anaphylaxis and to identify factors associated with elevated tryptase levels.

## Methods

Children presenting with anaphylaxis to the Montreal Children’s Hospital Emergency Department (ED) between April 2011 and April 2013 were recruited. The treating physician documented symptoms, triggers, and management of the anaphylactic reactions. Total tryptase levels were measured 30-120 minutes following onset of symptoms. Charts of all ED patients were reviewed to identify cases that were missed in prospective recruitment. Multivariate logistic regression was used to examine the association between an elevated tryptase level and age, gender, reaction trigger, reaction severity, and history of atopy.

## Results

Of 398 anaphylaxis cases (203 of whom were recruited prospectively), 84 children had serum tryptase levels measured. Age, gender, anaphylactic trigger, and severity of reaction were comparable between cases with and without tryptase measurements. However, there was higher percentage of patients treated with epinephrine in hospital in the group with tryptase measurements. The median age of these 84 children was 5.1 years (IQR 1.3, 12.3), 40.4% were females, 78.6% identified food as the potential anaphylactic trigger, and 7.1% experienced a severe reaction.. The mean tryptase level was 6.9 ng/mL (4.5, 8.0). Only 13 patients [15.5% (95%CI, 8.8,25.4)] had elevated levels. Severe reactions and history of eczema were associated with elevated levels ( OR =115.4 (95%CI,8.7,1527.8) and 14.2(95%CI,2.6,78.5) respectively.

## Conclusions

Our results do not support the use of total tryptase as a diagnostic tool in children with anaphylaxis. Given the poor sensitivity (13/84 = 15%) of the current tryptase threshold, new laboratory tests need to be developed to help establish the diagnose of anaphylaxis accurately. Severe reactions and presence of eczema are associated with high levels, but wide confidence intervals preclude definitive conclusions for the other risk factors we investigated.

